# Protein Timing Does Not Affect Next-Day Recovery of Strength or Power but May Enhance Aerobic Adaptations to Short-Term Variable Intensity Exercise Training in Recreationally Active Males: A Pilot Study

**DOI:** 10.3389/fspor.2020.568740

**Published:** 2020-10-22

**Authors:** Sarkis J. Hannaian, Mark N. Orlando, Sidney Abou Sawan, Michael Mazzulla, Daniel W. D. West, Daniel R. Moore

**Affiliations:** Faculty of Kinesiology and Physical Education, University of Toronto, Toronto, ON, Canada

**Keywords:** protein supplement, high intensity interval training (HIIT), muscle recovery, nutrient timing, exercise performance

## Abstract

**Background:** Variable intensity training (VIT) characteristic of stop-and-go team sport exercise may reduce performance capacity when performed on successive days but also represent a strategy to induce rapid training-induced increases in exercise capacity. Although post-exercise protein enhances muscle protein synthesis, the timing of protein ingestion following variable intensity training (VIT) on next-day recovery and short-term performance adaptation is unknown.

**Purpose:** To determine if immediate (IMM) as compared to delayed (DEL) protein ingestion supports greater acute recovery of exercise performance during successive days of VIT and/or supports chronic training adaptations.

**Methods:** Sixteen habitually active men performed 5 consecutive days of variable intensity training (VIT) in the evening prior to consuming a beverage providing carbohydrate and whey protein (IMM; 0.7 g and 0.3 g/kg, respectively) or carbohydrates alone (DEL; 1 g/kg) with the reciprocal beverage consumed the following morning. Performance was assessed before each VIT (recovery) and 2 days after the final VIT (adaptation).

**Results:** Five consecutive days of VIT progressively decreased anaerobic peak power (~7%) and muscle strength (MVC; ~8%) with no impact of protein timing. Following 2 days of recovery, VIT increased maximal voluntary contraction and predicted VO_2peak_ by ~10 and ~5%, respectively, with a moderate beneficial effect of IMM on predicted VO_2peak_ (ES = 0.78).

**Conclusion:** Successive days of simulated team sport exercise decreases markers of next-day performance capacity with no effect of protein timing on acute recovery. However, practical VIT increases muscle strength and aerobic capacity in as little as 5 days with the latter potentially enhanced by immediate post-exercise protein consumption.

## Introduction

Repeated bouts of variable intensity exercise are common training and/or competition demands of many team-sport athletes, and may result in performance decrements such as reduced maximal strength and anaerobic power when performed on consecutive days (Silva et al., 2018). While this reduction in performance may be mediated by exercise-induced muscle damage and/or neuromuscular fatigue (Twist and Eston, 2005), its rapid resolution could ultimately permit a greater training stimulus or performance capacity during a subsequent practice or competition. Protein consumption is essential to stimulate post-exercise muscle protein synthesis rates as a means to facilitate recovery (Moore, 2015). This has contributed to general recommendations for dietary protein for active populations (1.2–2.0 g·kg·d^−1^) (Thomas et al., 2016), with suggestions that supplementation may be important to sustain performance during periods of increased exercise volume and/or repeated exercise stimuli (e.g., training) (Pasiakos et al., 2014; Heaton et al., 2017), although current evidence is equivocal on its efficacy for team-sport athletes (Poulios et al., 2019).

The timing of post-exercise protein consumption is a popular and pragmatic approach designed to accelerate recovery (Schoenfeld et al., 2013). This involves consuming protein in and around a training session in an effort to acutely enhance the repair and remodeling of skeletal muscle proteins, thereby augmenting the skeletal muscle adaptive response. However, most studies investigating the impact of protein supplementation after real or simulated sports include a protein-free placebo and therefore may be confounded by total supplemental protein (Pasiakos et al., 2014), which ultimately precludes the ability to determine the effect of protein timing *per se*. Protein ingestion after a strenuous evening training bout is effective at stimulating overnight muscle protein synthesis rates (Res et al., 2012) as well as improving whole-body anabolism and markers of muscle strength and power during the subsequent 24 h recovery period (West et al., 2017). As evening training/competition may coincide with peak performance capacity (Atkinson and Reilly, 1996) and is common for many team sports (both professional and recreational), additional research is required to elucidate the potential benefit of immediate protein consumption before the typical overnight fasting period to support post-exercise recovery and the maintenance of next-day exercise performance.

Real or simulated team sport exercise can also be viewed as a practical model of variable intensity training (VIT), which includes intervals of higher intensity exercise that may enhance anaerobic power and aerobic capacity (MacInnis and Gibala, 2017). There is growing interest in the potential for nutritional manipulation of high-intensity training adaptations (Forbes et al., 2020). Protein ingestion after high intensity, aerobic exercise can enhance myofibrillar protein remodeling (Coffey et al., 2011), plasma volume expansion (Goto et al., 2010), and markers of mitochondrial biogenesis (Rowlands et al., 2011; Hill et al., 2013), all of which could be important to support adaptations to high intensity exercise training. However, the effect of protein timing, matched for total protein, in response to acute and consecutive daily bouts of evening VIT has yet to be investigated. Therefore, the primary aim of this preliminary research was to assess the impact of protein timing on day-to-day recovery from VIT. We also examined the impact of protein timing on the acute adaptation to VIT. Given the importance of post-exercise dietary protein to support skeletal muscle remodeling, we hypothesized that consuming protein immediately compared to delayed after a bout of VIT would: (i) enhance next-day exercise performance over 5 days of successive bouts, and; (ii) enhance short-term anaerobic and aerobic exercise adaptations.

## Methods

### Participants

Sixteen healthy, recreationally active men provided written informed consent to a protocol approved by the Faculty of Kinesiology and Physical Education Ethics Review Committee at the University of Toronto and conducted in accordance with the Declaration of Helsinki. Participants were recruited from the local University of Toronto community between September 2015 and August 2017. To be eligible, participants must have been between the age of 18 and 35 years and be participating in recreational team sports ≥ 3 times a week. Participants also had to obtain a minimum aerobic fitness (i.e., predicted VO_2peak_ ≥ 46.8 ml·kg^−1^·min^−1^) on a practical, multi-stage fitness test (Léger et al., 1988) and be able to complete the Loughborough Intermittent Shuttle Test (LIST). Participants were excluded if they self-reported any use of anabolic or pro-hormones for the previous 6 months or creatine or beta-alanine supplementation for the previous 4 weeks. All participants were healthy based on the Physical Activity Readiness Questionnaire. Participants were instructed to refrain from: (i) any exercise outside of the study protocol, (ii) the ingestion of any supplements (e.g., protein, creatine) and alcohol for the duration of the study (iii) avoid any caffeine for at least 3 h before any exercise/testing; the latter was designed to minimize any disturbances participants may experience within the pilot study and may not directly influence the performance outcomes (Guest et al., 2018).

### Familiarization

After familiarization with all performance tests (detailed below), body composition was assessed by air displacement plethysmography (BOD-POD, COSMED USA Inc., Chicago, IL) prior to participants being randomized to one of two post-exercise nutrition groups: immediate (IMM: 20 ± 2 y, 73.9 ± 6.3 kg, 51.9 ± 3.4 ml·kg^−1^·min^−1^ VO_2peak_; means ± *SD*) or delayed (DEL: 21 ± 3 y, 72.2 ± 6.2 kg, 52.3 ± 3.7 ml·kg^−1^·min^−1^ VO_2peak_). An average of two equations were used to predict VO_2peak_ following the completion of the Beep Test (Léger et al., 1988; Flouris et al., 2005), due to concerns of reliability of the former equation (Flouris et al., 2005).

### Performance Testing

**Figure 1:**
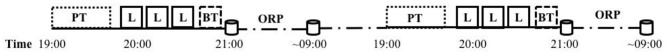
Experimental protocol schematic representing one 30-h period of the variable intensity training (VIT) week (i.e., 5 consecutive days). Performance testing (PT) consisted of (in sequence) squat jump height, maximal voluntary contraction, 30-s Wingate, and Beep Test (BT) to volitional fatigue. The VIT was a modified Loughborough Intermittent Shuttle Test (L; 3 × 15 min blocks across 60 min) (Packer et al., 2017). Immediately after the *L*, a task to failure (beep test; BT) was performed. Controlled beverages (cylinders) were consumed immediately after the BT (IMM: 0.7 g of protein and 0.3 g/kg of carbohydrates; DEL: 1 g/kg carbohydrates) and the following morning after the overnight recovery period (ORP; IMM: 1 g/kg carbohydrates; DEL: 0.7 g of protein and 0.3 g/kg of carbohydrates).

Performance testing occurred at the same time of day (19:00–21:00) in the same order (as listed below): tests were selected to measure outcomes that would be relevant for a team sport athlete including neuromuscular power (squat jump height; SJ), muscle strength (maximal voluntary contraction; MVC), anaerobic power (30-s Wingate), and aerobic capacity (Beep Test; BT), which with the exception of BT (Léger et al., 1988) have been described in detail previously (West et al., 2017). SJ was taken as the single best of three attempts on a force plate (Advanced Mechanical Technology Inc, Watertown, MA, USA). MVC (normalized to total body mass) was the highest isometric voluntary contraction of three attempts of the knee extensors on a custom dynamometer with force output signals recorded using PowerLab with LabChart Pro v.8.0.5 (ADInstruments Inc., Colorado Springs, CO, USA). Anaerobic mean power and peak power (PP) was assessed with a 30-s maximal Wingate test on a cycle ergometer with resistance equivalent to 7.5% of the participant's bodyweight (Monark Anaerobic Test Software v3.3, Monark Exercise AB, Vansbro, Sweden). Performance tests were repeated before each successive VIT to assess 24 h recovery as well as 2d after the final bout to determine short-term training adaptations. A schematic of the experimental protocol is illustrated in Figure 1.

Following the battery of performance testing, participants then performed the 1st of 5 consecutive days of VIT based on the LIST; a variable intensity shuttle test that simulates team sports (Packer et al., 2017). Briefly, three 15-min blocks of LIST separated by 5 min of recovery (Packer et al., 2017) were performed prior to BT testing. Rating of perceived exertion (RPE) was measured via visual analog scale after each performance test and each block of the LIST (mean RPE at the end of block 3 of the LIST for all 5 days of testing; IMM = 68%; DEL 65%).

### Beverage Composition and Ingestion

Immediately after the LIST, participants consumed one of two isocaloric beverages designed to support post-exercise recovery, including stimulating muscle protein synthesis rates and resynthesizing muscle glycogen (Moore, 2015, 2019): IMM ingested 0.3 g·kgBW^−1^ of whey protein isolate and 0.7 g·kgBW^−1^ carbohydrate (CHO) immediately after evening exercise and 1 g·kgBW^−1^ of CHO the following morning upon waking. DEL ingested the same macronutrients but in reverse order such that only supplement timing was different between groups. Supplements were provided in-kind by Iovate Health Sciences and verified to contain the specific contents by their in-house methods. Randomization and supplement preparation was performed by a collaborator who was not involved in the study testing or analysis. Both groups ingested a protein-free cookie (~72 and ~28% energy from carbohydrate and fat, respectively) (Zello et al., 1990) following the evening exercise bout that contained, when combined to the beverage intakes, replenished the estimated energy expenditure during the LIST was estimated for each participant by an accelerometer (SenseWear ProArmband, BodyMedia, Pittsburgh, PA) during the exercise familiarization session. Evening and morning supplements were consumed daily during 7d protocol including the 2d of recovery. Blinding was assessed by a brief exit questionnaire and revealed that less than half (7/16 participants) correctly guessed the group allocation demonstrating the efficacy of the blinding protocol.

### Energy and Macronutrient Intake

Participants were asked to record a 3-days weighed food log using a web-based platform (www.MyFitnessPal.com) prior to the training week to obtain an estimate of habitual dietary habits. In addition, participants completed 24-h food logs on three separate occasions during the training week in effort to estimate participants' relative compliance to habitual patterns of consumption. One participant from each group did not record dietary intakes during the 5-days. VIT therefore was not included in this analysis.

### Statistical Analyses

Changes in performance during consecutive days of VIT and before/after training were assessed using two-way ANOVA (group × day and group × training, respectively) with Sidak *post-hoc* (IBM SPSS Statistics Version 24), significance set at *P* < 0.05. Normality was assessed by Shapiro-Wilk test with a Wilcoxon and Friedman non-parametric test for data not normally distributed. *T*-tests were used to compare dietary intakes between groups. Cohen's *d* effect sizes (IMM vs. DEL) were calculated, with Cohen's *d* thresholds of small = 0.2, medium = 0.5, large = 0.8, and very large = 1.3. Data are reported as mean ± *SD*.

## Results

### Energy and Macronutrient Intake

There were no differences in total energy, protein, carbohydrate, and fat ingestion relative to body mass between IMM and DEL before (*P* ≥ 0.55) and during (*P* ≥ 0.61) training (Table 1).

**Table 1:** Energy and macronutrient intake*^a^*.

**Characteristic**	**Before^b^**		**During^c^**	
	**IMM (*n* = 8)**	**DEL (*n* = 8)**	**IMM (*n* = 7)**	**DEL (*n* = 7)**
Protein, g·kg^−1^·d^−1^	1.3 ± 0.4	1.5 ± 0.9	1.4 ± 0.5	1.4 ± 0.7
Protein, % energy	17 ± 3	20 ± 4	16 ± 2	17 ± 3
Carbohydrate, g·kg^−1^·d^−1^	3.8 ± 1.5	4.0 ± 1.7	5.1 ± 1.1	4.6 ± 1.2
Carbohydrate, % energy	50 ± 4	55 ± 10	59 ± 3	62 ± 12
Fat, g·kg^−1^·d^−1^	1.0 ± 0.5	1.0 ± 0.6	1.0 ± 0.3	0.9 ± 0.4
Fat, % energy	31 ± 5	30 ± 8	26 ± 4	26 ± 3
Energy, kcal·kg^−1^	30 ± 12	29 ± 12	34.5 ± 8	31.5 ± 11

a *Values are mean ± SD*.

b *Dietary intake before study based on 3 days weighed food log record*.

c *Dietary intake during study based on 3 × 24 h diet recall analysis (MyFitnessPal)*.

### Performance Recovery

**Figure 2:**
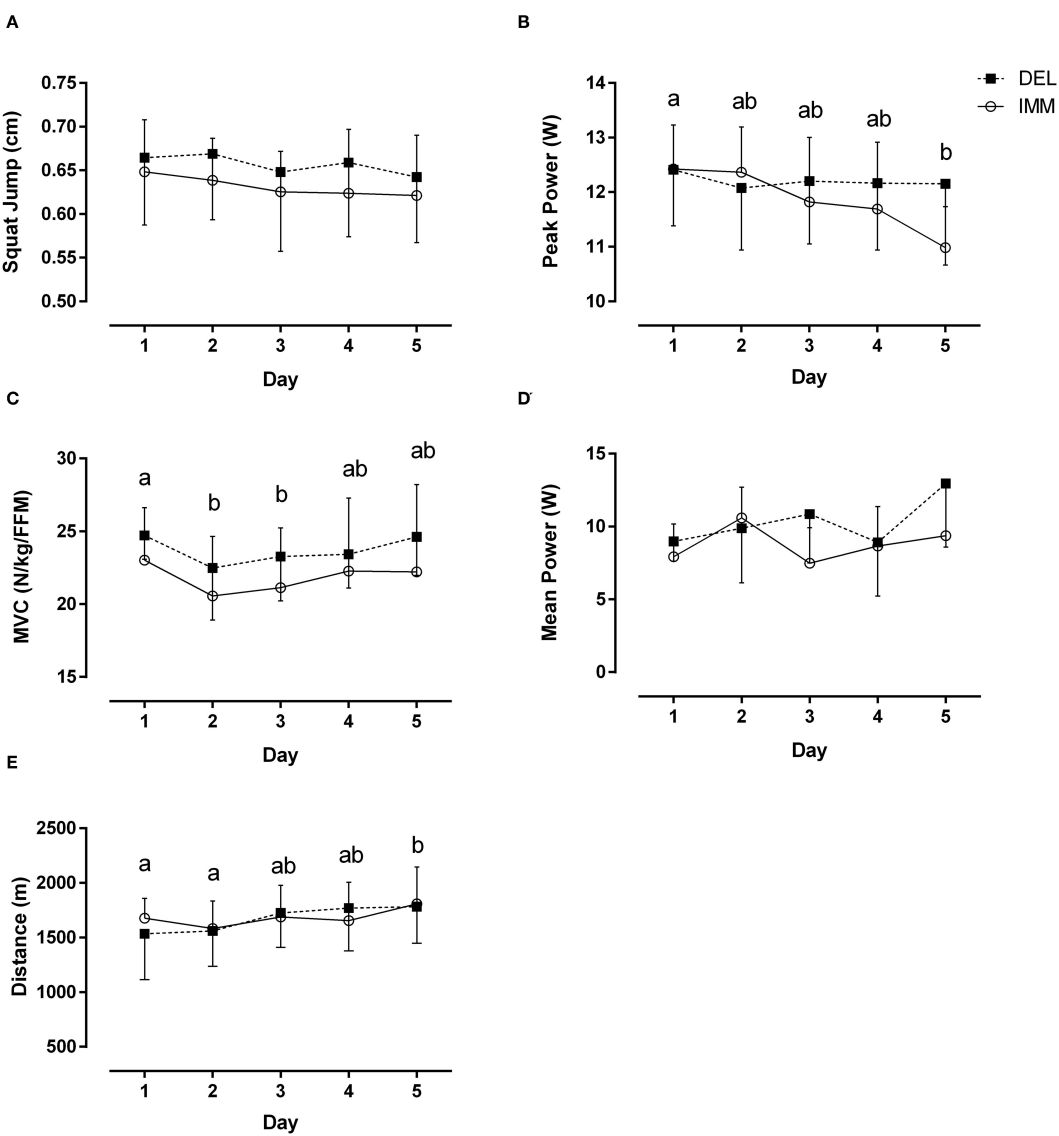
Day to day performance changes in **(A)** squat jump, **(B)** peak anaerobic power **(C)** MVC normalized to fat-free mass (FFM), **(D)** mean anaerobic power, and **(E)** distance traveled during multi-stage beep test on successive days of VIT in the evening after consuming protein immediately after (IMM) or the following morning (DEL) after exercise. Means with different letters are significantly different (main effect for time, *P* < 0.01). Mean ± *SD*.

There were no group by time interactions (both, *P* ≥ 0.09) for SJ and anaerobic mean power across the 5 days of exercise and recovery (Figures 2A,D). Anaerobic PP was decreased (main time effect, *P* < 0.01) at day 5 whereas MVC was transiently decreased (main time effect, *P* < 0.01) on days 2 and 3 (Figures 2B,C). Anaerobic PP was ~13% greater in DEL compared to IMM day 5, although this did not reach significance (*P* = 0.07). As the week progressed, the distance traveled during BT increased (main time effect, *P* < 0.05) ~12% from day 1 to 5 with no between-group differences (Figure 2E). There was no change (both, *P* ≥ 0.18) in SJ and anaerobic mean power after training

### Training Adaptation

**Figure 3:**
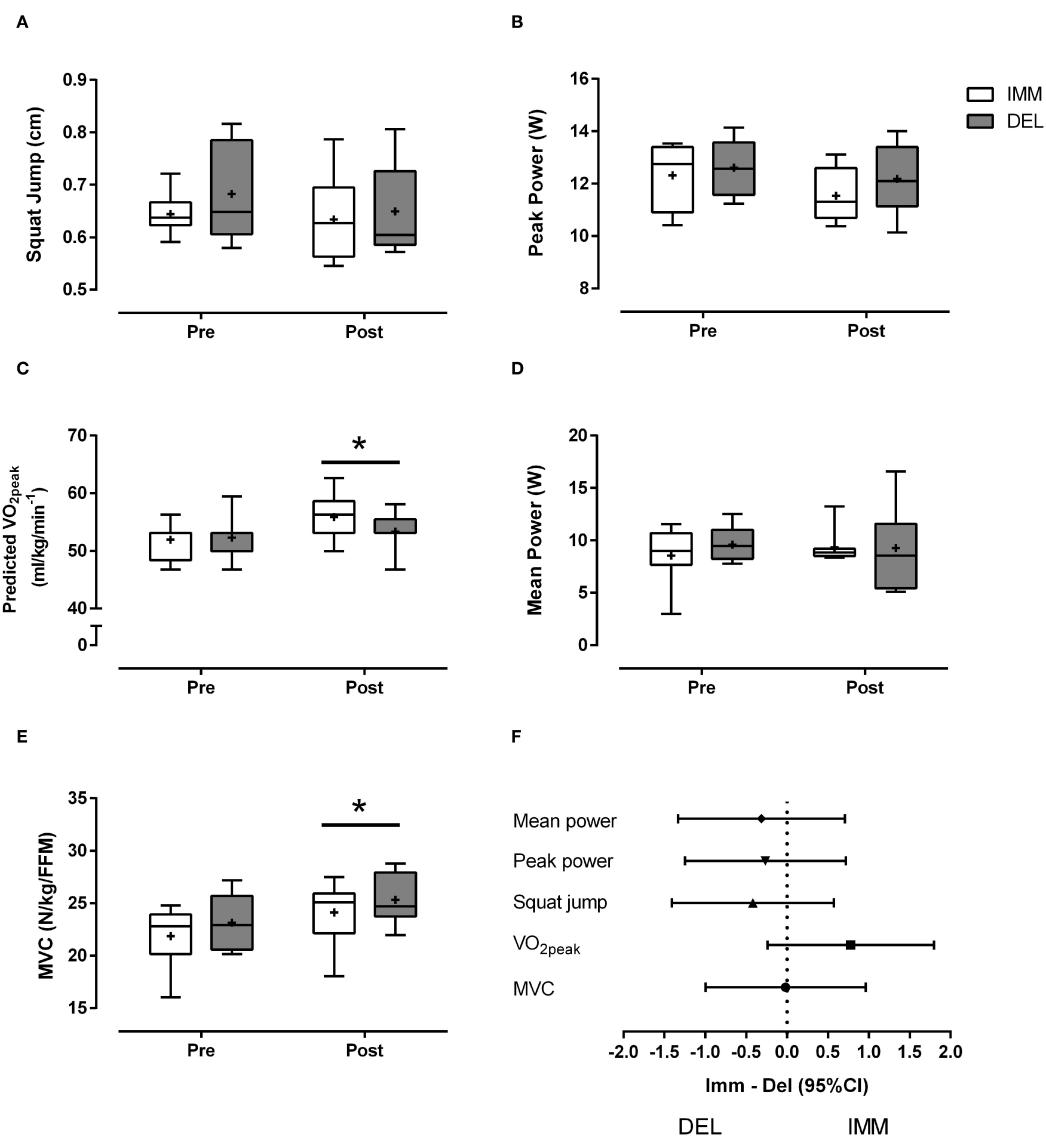
Box and whisker plot of **(A)** squat jump, **(B)** peak power **(C)** predicted VO_2peak_ **(D)** mean anaerobic power, and **(E)** MVC normalized to fat-free mass (FFM) before (Pre) and after (Post) 5 days of evening variable intensity exercise with protein consumption immediately after exercise (IMM) or the following morning (DEL). “Post” refers to testing that occurred 2 days after the final exercise bout. The horizontal line within the box represent the median, the boundaries around the box represent the 25 and 75th percentile, the whiskers indicate the minimum and maximum values, and the “+” marked in the box represent the mean. *different from pre (*P* < 0.05). Mean ± *SD*. **(F)** Effect sizes (Cohen's *d*: small = 0.2–0.5; moderate = 0.5–0.8; large = >0.8) and 95% confidence intervals for the mean effect of IMM– DEL on performance outcomes.

One week of training induced non-significant (main time effect, *P* = 0.08) decreases in anaerobic PP in IMM (~8%) and DEL (~7%) (Figure 3B). In contrast, there was an increase (main time effect, *P* < 0.01) in MVC for IMM (~10%) and DEL (~12%) (Figure 3E) and ~8% increase (main time effect, *P* < 0.05) for distance traveled in the BT (IMM: pre = 1,703 ± 261 m, post = 1,943 ± 238 m; DEL: pre = 1,663 ± 244 m, post = 1,835 ± 306 m). Training increased (main time effect, *P* = 0.02) predicted VO_2peak_ (Figure 3C) in IMM (~8%) and DEL (~2%) with a moderate effect size in favor of IMM (ES ± 95% CI: 0.78 ± 1.02) (Figure 3F). There were no differences observed in SJ performance and mean power on the Wingate following training (Figures 3A,D).

## Discussion

High-intensity sport-specific exercise is associated with acute decreases in exercise performance (e.g., strength loss, reduced sprint time, and/or vertical jump), which may be indicative of incomplete recovery (Doeven et al., 2018). In agreement with these findings, our study in recreationally active men observed a transient decrease (days 2 and 3) in muscle strength and a decrease in anaerobic peak power at day 5, although estimates of neuromuscular performance (i.e., SJ) were unaltered. We did not observe an effect of protein timing across the 5 days of exercise, suggesting previous benefits of protein supplementation on next-day exercise performance may be related in part to a greater total protein intake rather than its post-exercise ingestion *per se* (West et al., 2017). However, there is some evidence that protein supplementation up to 24 h after (but not before) exercise may improve indices of performance recovery (i.e., peak torque and reactive strength) after a single bout of damaging exercise (Cockburn et al., 2010). Thus, given the lack of a protein-free control group in the present study we cannot rule out the possibility that the protein supplement, regardless of timing, helped facilitate recovery in both groups, as has been suggested to occur with repeated protein supplementation and training (Pasiakos et al., 2014).

There was an increase in predicted VO_2peak_ after 1 week of VIT training, which could be consistent with the ability of high intensity training to induce aerobic adaptations over short periods (Krustrup et al., 2010; MacInnis and Gibala, 2017). The average ~5 and ~12% increases in predicted aerobic capacity and shuttle test distance, respectively, in only 5 days compares favorably with the ~8 and 37% improvements, respectively, that were previously reported after 12 weeks of 2–3 soccer matches per week (Krustrup et al., 2010). The relatively rapid increases in performance capacity would be broadly consistent with the rapid upregulation of mitochondrial enzymes reported after only six sessions of supramaximal sprint training (Burgomaster et al., 2005). Interestingly, these improvements in predicted VO_2peak_ may have been aided by immediate post-exercise protein ingestion, as suggested by the moderate effect in favor of immediate compared to delayed protein ingestion with *post-hoc* power analysis suggesting *n* = 13 per group would be required to see significance. Moreover, the increase in VO_2peak_ for IMM (~7.5%) was above the common retest variability reported previously in young males (i.e., ~5%) after appropriate familiarization (Cooper et al., 2005), which we interpret to reflect the increase in aerobic capacity was likely to be a general training response. There is some support for post-exercise and/or pre-sleep protein ingestion to enhance markers of aerobic adaptation (e.g., PGC-1α mRNA expression) (Hill et al., 2013) and capacity after endurance exercise (Knuiman et al., 2019), although this is not a universal finding (Jonvik et al., 2019). The potential mechanism(s) by which enhanced aerobic capacity may have been facilitated with immediate post-exercise protein ingestion are beyond the scope of the present study but could involve hematological adaptations (e.g., increased plasma volume) (Goto et al., 2010) and/or enhanced mitochondrial protein remodeling and enzyme expression (Rowlands et al., 2011).

We also observed an increase in MVC, which may be consistent with an ability of short term high intensity interval training to increase motor unit recruitment and muscle strength (Valdes, 2017). Insofar as enhanced myofibrillar remodeling may support strength adaptions, the ability of high force muscle contractions to enhance dietary amino acid sensitivity for up to 24 h could have rendered the issue of protein timing as being less relevant for this outcome (Burd et al., 2011; Schoenfeld et al., 2013). However, we (Ford et al., 2020) and others (Damas et al., 2018) have also reported no relationship between integrated rates of myofibrillar protein synthesis and muscle strength after damaging exercise and/or in response to acute resistance exercise (Ford et al., 2020), suggesting protein timing may not mediate the effects of VIT. However, it is also possible that the change in strength was a function of the repeated MVC measures over the 5-days protocol that facilitated a neuromuscular learning effect for this outcome (Mattocks et al., 2017), in which protein timing is unlikely to markedly influence.

We had speculated that the novel exercise bout including high force shortening (e.g., sprinting) and lengthening (e.g., deceleration) contractions would be associated with reductions in rapid force generation and neuromuscular function (Silva et al., 2013), especially given that the novel bouts were performed on consecutive days. However, this was not the case, with neuromuscular outcomes (SJ and PP) (Roe et al., 2017) neither decreasing in day-to-day performance nor increasing after the adaptation period. The lack of adaptation in both immediate and delayed protein ingestion may be related in part to: (i) the LIST being an insufficient stimulus to enhance neuromuscular adaptation in prime movers (e.g., hip and ankle extensors); (ii) the short duration (i.e., 7 days) of training being insufficient to alter muscle fiber size and/or heavy chain expression (Krustrup et al., 2010; Jakobsen et al., 2012), and/or; (iii) inadequate time to recover from the last training bout. To the latter point, previous literature has demonstrated that a single session of LIST impairs neuromuscular parameters up to 72 h (Magalhães et al., 2010), suggesting the non-significant ~7% decrease in anaerobic PP may be related in part to residual neuromuscular fatigue that compromised activity patterns in motor units responsible for high rate of force development movements (Mendez-Villanueva et al., 2008). Further, whereas protein supplementation has been reported to improve neuromuscular performance recovery after a single acute bout of exercise (Cockburn et al., 2010; West et al., 2017), data from the present study suggests that protein supplement timing may have limited scope for improving neuromuscular adaptation over consecutive bouts of exercise. However, it is possible that monitoring of other time-dependent variables of the SJ could have provided more insight into the potential for protein timing and/or VIT to influence neuromuscular fatigue (Gathercole et al., 2015).

Due to logistical challenges and the desire to perform a more ecologically valid “free-living” study, we did not include dietary controls during the training and recovery, which could be viewed as a potential limitation given the impact habitual intakes may have on the efficacy of protein supplementation (Pasiakos et al., 2014). Although web-based applications are effective at estimating habitual energy and macronutrient intakes, they, like other approaches, may be prone to under-reporting (Teixeira et al., 2018). Nevertheless, participants reported consuming ~1.4 g/kg protein during the VIT period while also supplementing with 0.3 g/kg whey protein, which collectively would have been sufficient to maximize protein synthesis with this type of training (i.e., >1.4 g/kg/d) (Packer et al., 2017). Moreover, this recommended protein intake for individuals performing team-sport type exercise represents ~15% of energy intake (Packer et al., 2017); this compares reasonably with participants in the present study (~16%), average males (~15%) (Lieberman et al., 2020), and well-trained team-sport athletes (~16%) (Gillen et al., 2017), all of which are within the Acceptable Macronutrient Distribution Range for protein (Wolfe et al., 2017). Importantly, no differences were observed between immediate and delayed protein ingestion in relative or percent energy for protein ingestion during the training week, which permitted us to determine the effect of supplement timing independent of total protein intake, a common limitation in previous studies. Our decision to provide evening and morning supplements—but not a fully controlled diet—was predicated on the desire to increase the ecological validity of the study. Further, we reasoned that protein supplementation (especially immediately post-exercise) may still facilitate recovery and enhance performance despite diet variation, including if diets contained suboptimal energy and/or protein intake (Pasiakos et al., 2014). Although we cannot discount the potential type II error in our pilot study for outcomes that were not affected by protein timing, we believe habitual dietary intake did not substantially influence the apparent benefit of immediate protein ingestion to enhance aerobic capacity. Nevertheless, future studies evaluating the potential impact of protein timing on post-exercise performance recovery and/or training adaptation could benefit from more stringent dietary controls.

## Conclusion

The present study provides insight on the effects of protein timing on subsequent day-to-day recovery and performance adaptation following VIT. We demonstrate that a practical VIT protocol that simulates team sport exercise transiently impairs next-day muscle strength and anaerobic peak power but improves predicted aerobic capacity and muscle strength in as little as 5 days in recreationally active young males. Future research would be beneficial to confirm the potential for immediate post-exercise protein consumption to enhance these aerobic adaptations, especially if exercise is performed in the evening.

## Data Availability Statement

The raw data supporting the conclusions of this article will be made available by the authors, without undue reservation.

## Ethics Statement

The studies involving human participants were reviewed and approved by University of Toronto Health Sciences Research Ethics Board. The patients/participants provided their written informed consent to participate in this study.

## Author Contributions

DM, DW, SH, and MO designed the study. SH, MO, SA, and MM carried out the study. SH, DW, SA, and MM performed statistical analysis. SH, DW, and DM interpreted the data. SH and DM wrote the manuscript. All authors read and approved the final manuscript.

## Conflict of Interest

The authors declare that the research was conducted in the absence of any commercial or financial relationships that could be construed as a potential conflict of interest.

## Abbreviations

BT: Beep Test
CHO: carbohydrate
DEL: delayed
ES: effect size
IMM: immediate
LIST: Loughborough Intermittent Shuttle Test
MVC: maximal voluntary contraction
PP: peak power
RPE: rating of perceived exertion
SJ: squat jump height
VIT: variable intensity training.
